# Supplementary material for: The efficacy of interventions in the workplace promoting exercise and a healthy diet among shift workers: A systematic review
Source: PLoS One. 2025 Jun 11;20(6):e0325071. doi: 10.1371/journal.pone.0325071 (PMC12157753; doi:10.1371/journal.pone.0325071)
Supplement: S1 Data — This work was supported by Research Financing and Incen-tive (Fipe) from Hospital de Clínicas de Porto Alegre (HCPA). (DOCX) [file pone.0325071.s001.docx]

| **Name of data extractors** | **Date of data extraction** |
| --- | --- |
| Francielle  Lopes dos  Reis | January to  December  2023 |
| Julio Cesar Ferreira  Bertoloto |  |
| Maria Carlota Borba Brum revisora |  |

1

Effects of a multi-behavioral health promotion program at worksite on smoking patterns and quit behavior.

Mache S; Vitzthum K; Groneberg DA; Harth V

2019-01-01



Do overweight workers profit by workplace health promotion, more than their normal-weight peers? Evaluation of a worksite intervention.

Mache S; Jens

3

A Simple Liking Survey Captures Behaviors Associated with Weight Loss in a Worksite Program among Women at Risk of Type 2 Diabetes.

Sharafi M; Faghri P; Huedo-Medina TB; Duffy VB

2021-04-17

4

Impact of a Worksite Diabetes Prevention Intervention on Diet Quality and Social Cognitive Influences of Health Behavior: A Randomized Controlled Trial.

Miller CK; Weinhold KR; Nagaraja HN

2016-03-01

5

Go!: results from a quasi-experimental obesity prevention trial with hospital employees.

LaCaille LJ; Schultz JF; Goei R; LaCaille RA; Dauner KN; de Souza R; Nowak AV; Regal R

2016-02-19

6

The integration of studio cycling into a worksite stress management programme.

Clark MM; Soyring JE; Jenkins SM; Daniels DC; Berkland BE; Werneburg BL; Hagen PT; Lopez-Jimenez F; Warren BA; Olsen KD

2014-04-01

7

The effectiveness and barriers of implementing a workplace health promotion program to improve metabolic disorders in older workers in Taiwan.

Chen MM; Tsai AC; Wang JY

2016-06-01

8

Impact of a group-based intervention program on physical activity and health-related outcomes in worksite settings.

Gu M; Wang Y; Shi Y; Yu J; Xu J; Jia Y; Cheng M

2020-06-15

9

Evaluation of a Voluntary Worksite Weight Loss Program on Metabolic Syndrome.

Earnest CP; Church TS

2015-11-01

10

A Computerized Lifestyle Application to Promote Multiple Health Behaviors at the Workplace: Testing Its Behavioral and Psychological Effects.

Lippke S; Fleig

11

Promoting changes in obesogenic behaviors: does coworker social support play a role?

Tamers SL; Thompson B; Cheadle AD; Zheng Y; Bishop SK; Beresford SA

2015-05-01

12

Effective weight-loss using an e-health delivered physical activity and dietary intervention: A federal credit union pilot study.

Gregoski MJ;

13

A Randomized Controlled Trial Translating the Diabetes Prevention Program to a University Worksite, Ohio, 2012-2014.

Weinhold KR; Miller CK; Marrero DG; Nagaraja HN; Focht BC; Gascon GM

2015-11-25

14

Worksite neighborhood and obesogenic behaviors: findings among employees in the Promoting Activity and Changes in Eating (PACE) trial.

Barrington WE; Beresford SA; Koepsell TD; Duncan GE; Moudon AV

2015-01-01

15

Process evaluation of a multifaceted health program aiming to improve physical activity levels and dietary patterns among construction workers.

Viester L; Verhagen EA; Bongers PM; van der Beek AJ

2014-11-01

16

Workplace exercise for changing health behavior related to physical activity.

Grande AJ; Cieslak F; Silva V

2015-01-01

17

A cluster randomized trial of alcohol prevention in small businesses: a cascade model of help seeking and risk reduction.

Reynolds GS; Bennett JB

2015-01-01

18

Effect of a 5-Month Worksite Physical Activity Program on Tertiary Employees Overall Health and Fitness.

Genin PM; Degoutte F; Finaud J; Pereira B; Thivel D; Duclos M

2017-02-01

19

Implementation of an Internet Weight Loss Program in a Worksite Setting.

Ross KM; Wing RR

2016-01-01

20

Improving employee health: evaluation of a worksite lifestyle change program to decrease risk factors for diabetes and cardiovascular disease.

Kramer MK; Molenaar DM; Arena VC; Venditti EM; Meehan RJ; Miller RG; Vanderwood KK; Eaglehouse Y; Kriska AM

2015-03-01

21

Weight-Dependent Disparities in Adolescent Girls: The Impact of a Brief Pilot Intervention on Exercise and Healthy Eater Identity.

Kramer EN; Chard CA; Walters K; Barr-Anderson DJ

2018-07-04

22

The Impact of a Worksite-Based Diabetes Prevention Intervention: A Pilot Study.

Clark B; Boghani S; Grullon C; Batista M

2017-06-01

23

Promoting physical activity in worksite settings: results of a German pilot study of the online intervention Healingo fit.

Dadaczynski K; Schiemann S; Backhaus O

2017-09-08

24

Worksite Food and Physical Activity Environments and Wellness Supports Reported by Employed Adults in the United States, 2013.

Onufrak S

25

Ferrari Corporate Wellness Program: Results of a Pilot Analysis and the "Drag" Impact in the Workplace.

Biffi A; Fernando F; Adami PE; Messina M; Sirico F; Di Paolo F; Coluccia R; Borghi C; D'Ascenzi F; Volpe M

2018-09-01

26

Worksite influences on obesogenic behaviors in low-wage workers in St Louis, Missouri, 2013-2014.

Strickland JR; Pizzorno G; Kinghorn AM; Evanoff BA

2015-05-07

27

Worksite Physical Activity Intervention for Ambulatory Clinic Nursing Staff.

Tucker S; Farrington M; Lanningham-Foster LM; Clark MK; Dawson C; Quinn GJ; Laffoon T; Perkhounkova Y

2016-07-01

28

The Impact of a Policy-Based Multicomponent Nutrition Pilot Intervention on Young Adult Employee's Diet and Health Outcomes.

Schliemann D; McKinley M; Woodside JV

2019-03-01

29

A nutritional intervention programme at a worksite canteen to promote a healthful lifestyle inspired by the traditional Mediterranean diet.

Vitale M; Bian

30

The Effectiveness of a Worksite Lifestyle Intervention Program on High-Risk Individuals as Potential Candidates for Bariatric Surgery: My Unlimited Potential (MyUP).

Osondu CU; Aneni EC; Shaharyar S; Roberson L; Rouseff M; Das S; Spatz E; Younus A; Guzman H; Brown D; Santiago-Charles J; Ochoa T; Mora J; Gilliam C; Lehn V; Sherriff S; Tran T; Gonzalez A; Virani S; Feldman T; Agatston AS; Nasir K

2016-10-01

31

Evaluation of a Digital Behavioral Counseling Program for Reducing Risk Factors for Chronic Disease in a Workforce.

Wilson MG; Castro Sweet CM; Edge MD; Madero EN; McGuire M; Pilsmaker M; Carpenter D; Kirschner S

2017-08-01

32

Changing Diet and Physical Activity in Nurses: A Pilot Study and Process Evaluation Highlighting Challenges in Workplace Health Promotion.

Torquati L; Kolbe-Alexander T; Pavey T; Leveritt M

2018-11-01

33

A Cluster Randomized Controlled Trial of a Total Worker Health(®) Intervention on Commercial Construction Sites.

Peters SE; Grant MP; Rodgers J; Manjourides J; Okechukwu CA; Dennerlein JT

2018-10-25

34

Make a Move Intervention to Reduce Childhood Obesity.

Nerud K; Samr

35

Evaluation of Get Healthy at Work, a state-wide workplace health promotion program in Australia.

Crane M; Bohn-Goldbaum E; Lloyd B; Rissel C; Bauman A; Indig D; Khanal S; Grunseit A

2019-02-13

36

Baton Rouge Healthy Eating and Lifestyle Program (BR-HELP): A Pilot Health Promotion Program.

Kennedy BM; Ryan DH; Johnson WD; Harsha DW; Newton RL Jr; Champagne CM; Allen HR; Katzmarzyk PT

2015-01-01

37

Workplace social and organizational environments and healthy-weight behaviors.

Tabak RG; Hipp JA; Marx CM; Brownson RC

2015-01-01

38

Health and Fitness Benefits But Low Adherence Rate: Effect of a 10-Month Onsite Physical Activity Program Among Tertiary Employees.

Genin PM; Dessenne P; Finaud J; Pereira B; Thivel D; Duclos M

2018-09-01

39

Eating Pleasure in a Sample of Obese Brazilian Women: A Qualitative Report of an Interdisciplinary Intervention Based on the Health at Every Size Approach.

Sabatini F; Ulian MD; Perez I; Pinto AJ; Vessoni A; Aburad L; Benatti FB; Lopes de Campos-Ferraz P; Coelho D; de Morais Sato P; Roble OJ; Unsain RF; Schuster RC; Gualano B; Scagliusi FB

2019-09-01

40

Occupational health and metabolic risk factors: A pilot intervention for transport workers.

Naug HL; Cols

41
Mechanisms of Physical Activity Behavior Change in an Incentive-Based Intervention: Mediation Analysis

Murray, JM; F

42

Can certified health professionals treat obesity in a community-based programme? A quasi-experimental study.

Miedema B; Reading SA; Hamilton RA; Morrison KS; Thompson AE

2015-02-04

43

Extent and correlates of change in anthropometric and fitness outcomes among participants in a corporate team-based weight loss challenge in Singapore: lose to win 2009.

Vasquez K; Malhotra R; Østbye T; Chan MF; Amin H; Khoo G; Choo L; Chew L; Thilagaratnam S

2015-03-01

44

Inducing a health-promoting change process within an organization: the effectiveness of a large-scale intervention on social capital, openness, and autonomous motivation toward health.

van Scheppingen AR; de Vroome EM; Ten Have KC; Bos EH; Zwetsloot GI; van Mechelen W

2014-11-01

45

The association between worksite physical environment and employee nutrition, and physical activity behavior and weight status.

Almeida FA; Wall SS; You W; Harden SM; Hill JL; Krippendorf BE; Estabrooks PA

2014-07-01

46

A Men's Workplace Health Intervention: Results of the POWERPLAY Program Pilot Study.

Johnson ST; Stolp S; Seaton C; Sharp P; Caperchione CM; Bottorff JL; Oliffe JL; Jones-Bricker M; Lamont S; Medhurst K; Errey S; Healy T

2016-08-01

47

Worksite health promotion program participation: a study to examine the determinants of participation.

Hall ME; Bergman RJ; Nivens S

2014-09-01

48

Comparative Effectiveness of Two Walking Interventions on Participation, Step Counts, and Health.

Smith-McLallen A; Heller D; Vernisi K; Gulick D; Cruz S; Snyder RL

2017-03-01

49

Assessing the Efficacy of a Group Mediated Nutritional Knowledge Intervention for Individuals with Obesity.

Miedema B; Bowes A; Hamilton R; Reading S

2016-12-01

50

The Effects of an Academic--Workplace Partnership Intervention to Promote Physical Activity in Sedentary Office Workers.

Chae D; Kim S

51

Associations of working from home with occupational physical activity and sedentary behavior under the COVID-19 pandemic

Fukushima, N; M

52

A randomized controlled trial to evaluate outcomes of a workplace self-management intervention and an intensive monitoring intervention.

Schopp LH; Clark MJ; Lamberson WR; Uhr DJ; Minor MA

2017-06-01

53

Changes in Men's Physical Activity and Healthy Eating Knowledge and Behavior as a Result of Program Exposure: Findings From the Workplace POWERPLAY Program.

Caperchione CM; Stolp S; Bottorff JL; Oliffe JL; Johnson ST; Seaton C; Sharp P; Jones-Bricker M; Lamont S; Errey S; Healy T; Medhurst K; Christian H; Klitch M

2016-12-01

54

Obesity Prevention Worksite Wellness Interventions for Health Care Workers: A Narrative Review.

Upadhyaya M; Sharma S; Pompeii LA; Sianez M; Morgan RO

2020-01-01

55

Impact of non-diet approaches on attitudes, behaviors, and health outcomes: a systematic review.

Clifford D; Ozier A; Bundros J; Moore J; Kreiser A; Morris MN

2015-03-01

56

Dietary and behavior changes following RDN-led corporate wellness counseling: A secondary analysis.

Hicks-Roof KK; Franklin MP; Sealey-Potts CV; Zeglin RJ

2021-01-01

1

57

A Minimal Contact Diet and Physical Activity Intervention for White-Collar Workers.

Gretebeck KA; Bailey T; Gretebeck RJ

2017-09-01

58

Pilot Investigation of 2 Nondiet Approaches to Improve Weight and Health.

Webber KH; Mellin L; Mayes L; Mitrovic I; Saulnier M

2018-01-01

59

Enhancing workplace wellness efforts to reduce obesity: a qualitative study of low-wage workers in St Louis, Missouri, 2013-2014.

Strickland JR; Eyler AA; Purnell JQ; Kinghorn AM; Herrick C; Evanoff BA

2015-05-07

60

Impact of a Weight Management Intervention on Eating Competence: Importance of Measurement Interval in Protocol Design.

Lohse B; Krall JS; Psota T; Kris-Etherton P

2018-03-01

61

A multicenter randomized controlled trial of a nutrition intervention program in a multiethnic adult population in the corporate setting reduces depression and anxiety and improves quality of life: the GEICO study.

Agarwal U; Mishra S; Xu J; Levin S; Gonzales J; Barnard ND

2015-03-01

62

Wellness programme at the workplace promotes dietary change and improves health indicators in a longitudinal retrospective study.

Soliman GA; Kim J; Lee JM; High R; Hortman S; Kim Y; Wehbi NK; Canedy J

2019-02-01

63

Effect of Workplace Counseling Interventions Launched by Workplace Health Promotion and Tobacco Control Centers in Taiwan: An Evaluation Based on the Ottawa Charter.

Chen TH; Huang JJ; Chang FC; Chang YT; Chuang HY

2016-01-01

64

The SHIELD (Safety & Health Improvement: Enhancing Law Enforcement Departments) Study: Mixed Methods Longitudinal Findings.

Kuehl KS; Elliot DL; MacKinnon DP; O'Rourke HP; DeFrancesco C; Miočević M; Valente M; Sleigh A; Garg B; McGinnis W; Kuehl H

2016-05-01

65

The practice of active rest by workplace units improves personal relationships, mental health, and physical activity among workers.

Michishita R; Jiang Y; Ariyoshi D; Yoshida M; Moriyama H; Yamato H

2017-03-28

66

The Effects of Two Workplace Weight Management Programs and Weight Loss on Health Care Utilization and Costs.

Østbye T; Stroo M; Eisenstein EL; Dement JM

2016-02-01

67

Lifestyle characteristics as moderators of the effectiveness of weight control interventions among semiconductor workers.

Lin TY; Liao PJ; Ting MK; Hsu KH

2018-12-01

data

68

Gamification of nutrition: A preliminary study on the impact of gamification on nutrition knowledge, attitude, and behaviour of adolescents in Nigeria.

Ezezika O; Oh J; Edeagu N; Boyo W

2018-09-01

69

Meal Planning Program to Reduce Barriers and Improve Diet Quality in Worksite Wellness Center Members.

Zeratsky KA; McMahon MM; Jenkins SM; Clark MM

2018-11-01

70

"A pilot study of the nutrition and exercise for wellness and recovery (NEW-R): A weight loss program for individuals with serious mental illnesses": Correction to Brown et al. (2015).

2015-12-01

71

Effectiveness of a healthcare-based mobile intervention on sedentary patterns, physical activity, mental well-being and clinical and productivity outcomes in office employees with type 2 diabetes: study protocol for a randomized controlled trial

Alòs, F; Colome

72

Effects of Obesity Management Program Provided by Occupational Health Nurse in Worksite.

Sok SR; Kim OS; Park MH

2019-05-01

73

Readiness for health behavior changes among low fitness men in a Finnish health promotion campaign.

Kaasalainen KS; Kasila K; Komulainen J; Malvela M; Poskiparta M

2016-12-01

74

Intervention fidelity and effectiveness of a UK worksite physical activity intervention funded by the BUPA Foundation, UK.

Lawton R; Mceachan R; Jackson C; West R; Conner M

2015-03-01

75

Effects of partners together in health intervention on physical activity and healthy eating behaviors: a pilot study.

Yates BC; Norman J; Meza J; Krogstrand KS; Harrington S; Shurmur S; Johnson M; Schumacher K

2015-03-01

76

[Effectiveness of physical activity intervention at workplace].

Malińska M

2017-03-24

77

Workplace exercise and educational program for improving fitness outcomes related to health in workers: a randomized controlled trial.

Vilela BL; Benedito Silva AA; de Lira CA; Andrade Mdos S

2015-03-01

78

Review of measures of worksite environmental and policy supports for physical activity and healthy eating.

Hipp JA; Reeds DN; van Bakergem MA; Marx CM; Brownson RC; Pamulapati SC; Hoehner CM

2015-05-07

1

79

The Centers for Disease Control and Prevention: Findings From The National Healthy Worksite Program.

Lang J; Cluff L; Payne J; Matson-Koffman D; Hampton J

2017-07-01

80 Intervention Strategies for the National Project of Workplace Health Promotion in China.

Li S; Li T; Li C

81

Evaluation of Worksite Wellness Nutrition and Physical Activity Programs and Their Subsequent Impact on Participants' Body Composition.

Sandercock V; Andrade J

2018-01-01

82

Is work keeping us from acting healthy? How workplace barriers and facilitators impact nutrition and exercise behaviors.

Mazzola JJ; Moore JT; Alexander K

2017-12-01

83

Dietary Interventions to Promote Healthy Eating among Office Workers: A Literature Review.

Glympi A; Chasioti A; Bälter K

2020-12-07

84

Post-traumatic stress disorder, physical activity, and eating behaviors.

Hall KS; Hoerste

85

Determining Barriers and Facilitators Associated With Willingness to Use a Personal Health Information Management System to Support Worksite Wellness Programs.

Neyens DM; Childers AK

2017-07-01

86

Reducing the risk of metabolic syndrome at the worksite: preliminary experience with an ecological approach.

Lucini D; Zanuso S; Solaro N; Vigo C; Malacarne M; Pagani M

2016-02-01

87

Employee Perceptions of Workplace Health Promotion Programs: Comparison of a Tailored, Semi-Tailored, and Standardized Approach.

Street TD; Lacey SJ

2018-04-28

88

Enhancing employee wellness: Translating an effective community behavioral weight-loss treatment to the worksite.

Murakami JM; Bennett BL; Rand-Giovannetti D; Stefano EC; Latner JD

2020-09-01

89

Short-Term Efficacy of a "Sit Less, Walk More" Workplace Intervention on Improving Cardiometabolic Health and Work Productivity in Office Workers.

Lin YP; Lin CC; Chen MM; Lee KC

2017-03-01

90

Perceived Barriers to Healthy Eating and Physical Activity Among Participants in a Workplace Obesity Intervention.

Stankevitz K; Dement J; Schoenfisch A; Joyner J; Clancy SM; Stroo M; Østbye T

2017-08-01

91

A multistage controlled intervention to increase stair climbing at work: effectiveness and process evaluation.

Bellicha A; Kieusseian A; Fontvieille AM; Tataranni A; Copin N; Charreire H; Oppert JM

2016-04-11

92

Effects of a Worksite Group Intervention to Promote Physical Activity and Health: The Role of Psychological Coaching.

Krebs S; Baaken A; Wurst R; Goehner W; Fuchs R

2019-11-01

93

Evaluation of a Worksite-Based Small Group Team Challenge to Increase Physical Activity.

Tullar JM; Wa

94

Effects of Obesity Management Program Provided by Occupational Health Nurse in Worksite.

Sok SR; Kim O

95

Increased Physical Activity Leads to Improved Health-Related Quality of Life Among Employees Enrolled in a 12-Week Worksite Wellness Program.

Macaluso S; Marcus AF; Rigassio-Radler D; Byham-Gray LD; Touger-Decker R

2015-11-01

96

The Effect of New Shower Facilities on Physical Activity Behaviors of Employees: A Quasi-experiment.

Nehme EK; Pérez A; Ranjit N; Amick BC 3rd; Kohl HW 3rd

2017-02-01

97

The Impact of a Workplace Wellness Program on Provider Health in Early Childhood Education Settings.

Powers JN; Farewell CV; Maiurro E; Puma J

2020-02-01

98

Moderators of intervention dose effects on diet quality and physical activity changes in a church-based, multicomponent, lifestyle study: Delta Body and Soul III.

Thomson JL; Zo

99

Healthy Lifestyle Behaviors and Health Promotion Attitudes in Preregistered Nurses: A Questionnaire Study.

Blake H; Stanulewicz N; Griffiths K

2017-02-01

100

Evaluation of health education interventions on Chinese factory workers' knowledge, practices, and behaviors related to infectious disease.

Li L; Xu W; Wagner AL; Dong X; Yin J; Zhang Y; Boulton ML

2019-01-01

101

Promotion of the mediterranean diet incancer long-survivors by means of the Med-Food Anticancer Program: a pilot study.

Panunzio M; Caporizzi R; Cela EP; Antoniciello A; Di Martino V; Ferguson LR

2019-01-01

102

Reach and effectiveness of an integrated community-based intervention on physical activity and healthy eating of older adults in a socioeconomically disadvantaged community.

Luten KA; Reijneveld SA; Dijkstra A; de Winter AF

2016-02-01

103

Testing the effect of text messaging cues to promote physical activity habits: a worksite-based exploratory intervention.

Fournier M; d'Arripe-Longueville F; Radel R

2017-10-01

104

A Million Steps: Developing a Health Promotion Program at the Workplace to Enhance Physical Activity.

González-Dominguez ME; Romero-Sánchez JM; Ares-Camerino A; Marchena-Aparicio JC; Flores-Muñoz M; Infantes-Guzmán I; León-Asuero JM; Casals-Martín F

2017-11-01

105

Stages of change of behavior in women on a multi-professional program for treatment of obesity.

Bevilaqua CA; Pelloso SM; Marcon SS

2016-10-10

106

["Fit4You"--A Programme for Prevention and Reduction of Overweight in Apprentices in the Workplace Setting].

Angerer P; Niedermeier H; Graf T; Manthey A; Marten-Mittag B; Schmidt HL; Gündel H

2015-09-01

107

Physical activity promotion in primary health care in Brazil: a counseling model applied to community health workers.

Florindo AA; Costa EF; Sa TH; dos Santos TI; Velardi M; Andrade DR

2014-11-01

108

Perceptions of a Peer Modeling Workplace Physical Activity Intervention for Women.

Rowland SA; Cohen MZ; Pullen CH; Schulz PS; Berg KE; Yates BC

2018-09-01

109

Implementation and Evaluation of a Physical Activity and Dietary Program in Federal Incarcerated Females.

Johnson RA; M

110

Modifiable Healthy Lifestyle Behaviors: 10-Year Health Outcomes From a Health Promotion Program.

Byrne DW; Rolando LA; Aliyu MH; McGown PW; Connor LR; Awalt BM; Holmes MC; Wang L; Yarbrough MI

2016-12-01

111

Make Your Move Experience: A Worksite Wellness Pilot in South Texas.

Wilkinson AV; Davé A; Ozdemir E; Rodriquez L; Reininger BM

2020-02-01

112

Greater Average Meal Planning Frequency Predicts Greater Weight Loss Outcomes in a Worksite-Based Behavioral Weight Loss Program.

Hayes JF; Balantekin KN; Fitzsimmons-Craft EE; Jackson JJ; Ridolfi DR; Boeger HS; Welch RR; Wilfley DE

2021-02-12

113

Effect of Intensity and Program Delivery on the Translation of Diabetes Prevention Program to Worksites: A Randomized Controlled Trial of Fuel Your Life.

Wilson MG; DeJ

114

Total Worker Health® Intervention for Construction Workers Alters Safety, Health, Well-Being Measures.

Anger WK; Kyler-Yano J; Vaughn K; Wipfli B; Olson R; Blanco M

2018-08-01

115

Age Differences in Health Behavior and Weight Changes in Japanese Workers: 1-Year Follow-Up Study.

Suka M; Yamauchi T; Yanagisawa H

2018-09-01

116

Improving employees' work-related well-being and physical health through a technology-based physical activity intervention: A randomized intervention-control group study.

Lennefer T; Lop

117

Building a Healthier Workforce: An Evaluation of an Online Nutrition Training for Apprentices.

Rohlman DS; Parish MA; Hanson GC; Williams LS

2018-10-01

118

DASH to wellness: emphasizing self-regulation through e-health in adults with prehypertension.

Dorough AE; Winett RA; Anderson ES; Davy BM; Martin EC; Hedrick V

2014-03-01

119

Challenging body weight: evidence from a community-based intervention on weight, behaviour and motivation.

Blais LT; Mack DE; Wilson PM; Blanchard CM

2017-08-01

120

[Perceived quality of life in the "healthy people" municipal community health promotion program in Ciudad Lineal-Madrid].

Esteban-Peña M; Gerechter-Fernández S; Martínez-Simancas AM; Zancada-González J; Hernandez-Barrera V; Jiménez-García R

2017-07-01

121

Results of an Academic, Health Care Worksite Weight Loss Contest for Southeastern Americans: Scale Back Alabama 2011-2013.

Breaux-Shropshire TL; Whitt L; Oster RA; Lewis D Jr; Shropshire TS; Calhoun DA

2015-04-01

122

Born Fat: The Relations Between Weight Changeability Beliefs and Health Behaviors and Physical Health.

Parent MC; Alquist JL

2016-06-01

123

Predictors of Health Promotion Behaviors Among Working Adults at Risk for Metabolic Syndrome.

Park S; Jang MK; Park CG; Hong OS

2022-07-01

124

The POWERPLAY workplace physical activity and nutrition intervention for men: Study protocol and baseline characteristics.

Caperchione CM; Sharp P; Bottorff JL; Stolp S; Oliffe JL; Johnson ST; Jones-Bricker M; Errey S; Christian H; Healy T; Medhurst K; Lamont S

2015-09-01

125

Feasibility of Online Nutrition Education in the Workplace: Working Toward Healthy Lifestyles.

Thomson JL; Go

126

Theory-Based, Participatory Development of a Cross-Company Network Promoting Physical Activity in Germany: A Mixed-Methods Approach.

Hoffmann C; Stassen G; Schaller A

2020-12-01

127

Evaluation of a Lifestyle Change Worksite Weight Management Program Across Multiple Employers and Sites.

Hales SB; Turner T; Sword DO; Nance L; Brown JD; O'Neil PM

2018-12-01

128

Testing a novel multicomponent intervention to reduce meat consumption in young men.

Amiot CE; El Hajj Boutros G; Sukhanova K; Karelis AD

2018-01-01

129

Improving sleep: outcomes from a worksite healthy sleep program.

Steffen MW; H

130

Participants' Perceptions of Worksite Health-Promotion Educational Activities.

Abell CH; Main ME

2016-10-01

131

The COMPASS pilot study: a total worker Health™ intervention for home care workers.

Olson R; Wright RR; Elliot DL; Hess JA; Thompson S; Buckmaster A; Luther K; Wipfli B

2015-04-01

132

Impact and Moderating Variables of an Intervention Promoting Physical Activity Among Children: Results From a Pilot Study.

Gourlan M; Takito M; Lambert C; Fregeac B; Alméras N; Coste O; Pereira B; Cousson-Gélie F

2018-04-01

133

Well-being, health, and productivity improvement after an employee well-being intervention in large retail distribution centers.

Rajaratnam AS; Sears LE; Shi Y; Coberley CR; Pope JE

2014-12-01

134

A pilot study using egocentric network analysis to assess holistic health benefits among a sample of university employees involved in a worksite fitness program.

Patterson MS; Gagnon LR; Nelon JL; Spadine MN

2020-01-01

135

A Move-A-Thon Event: A Workplace Demonstration of a Proposed Alternative Strategy to Incentivize Workers to Engage in Physical Activity.

Reutman S; Lewis R

2019-04-01

136

A 3-Year Workplace-Based Intervention Program to Control Noncommunicable Disease Risk Factors in Sousse, Tunisia.

Bhiri S; Maatoug J; Zammit N; Msakni Z; Harrabi I; Amimi S; Mrizek N; Ghannem H

2015-07-01

137

Implementing an Integrated Health Protection/Health Promotion Intervention in the Hospital Setting: Lessons Learned From the Be Well, Work Well Study.

Sorensen G; N

138

Changes in Fruit and Vegetable Consumption and Leisure Time Physical Exercise after a Citizen Science-Based Worksite Health Promotion Program for Blue-Collar Workers.

van der Feltz S; van der Molen HF; Lelie L; Hulshof CTJ; van der Beek AJ; Proper KI

2022-10-21

139

Blood Pressure Changes After a Health Promotion Program Among Mexican Workers.

Garcia-Rojas IJ; Omidakhsh N; Arah OA; Krause N

2021-01-01

140

Effects of a worksite physical activities program among men and women: An interventional study in a footwear industry.

Miranda Bispo

141

Health Promotion at the Construction Work Site: The Lunch Truck Pilot Study.

Caban-Martinez AJ; Moore KJ; Clarke TC; Davila EP; Clark JD 3rd; Lee DJ; Fleming LE

2018-12-01

142

Factors Associated With Interest in Worksite Health-Related Discussions/Events Among Employed Adults With Chronic Conditions.

Meng L; Galyardt AK; Robinson KT; DeJoy DM; Padilla HM; Zuercher H; Bien M; Smith ML

2017-07-01

143

Health Benefits of a 16-Week Whole Food, High Fiber, Plant Predominant Diet among U.S. Employees.

Kelly R; Hanus A; Payne-Foster P; Calhoun J; Stout R; Sherman BW

2023-02-01

1

144

Using Positive Nudge to Promote Healthy Eating at Worksite: A Food Labeling Intervention.

Montagni I; Prevot F; Castro Z; Goubel B; Perrin L; Oppert JM; Fontvieille AM

2020-06-01

145

[Effectiveness of Health Promotion Program Using Action Planning Strategy for Young Adults].

Kim SH; Kim MJ; Kim SH; Kim SY; Park CY; Bang JY

2019-08-01

146

Outcome-based and Participation-based Wellness Incentives: Impacts on Program Participation and Achievement of Health Improvement Targets.

Barleen NA; Marzec ML; Boerger NL; Moloney DP; Zimmerman EM; Dobro J

2017-03-01

147

A Structured Health Intervention for Truckers (SHIFT): A Process Evaluation of a Pilot Health Intervention in a Transport Company.

Varela-Mato V; Caddick N; King JA; Yates T; Stensel DJ; Nimmo MA; Clemes SA

2018-04-01

148

Attitudes Surrounding a Community-Based Fitness Intervention at an Urban FQHC.

Foster K; Stoeckle J; Silverio A; Castellan C; Hogue A; Gouch A; Weinstein L

2019-07-01

149

A Cross-Disciplinary Successful Aging Intervention and Evaluation: Comparison of Person-to-Person and Digital-Assisted Approaches.

Hsu HC; Kuo T; Lin JP; Hsu WC; Yu CW; Chen YC; Xie WZ; Hsu WC; Hsu YL; Yu MT

2018-05-04

150

A Worksite Weight Loss Program: An Innovative Way to Improve Obesity.

Mack A

2021-11-01

151

Evaluation of a successful aging promotion intervention program for middle-aged adults in Taiwan.

Hsu HC; Chuang SH; Hsu SW; Tung HJ; Chang SC; Lee MM; Wang JY; Kuo LT; Tseng FY; Po AT

2019-03-01

152

A 7-Step Strategy for the Implementation of Worksite Lifestyle Interventions: Helpful or Not?

Wierenga D; E

153

Impact Model-Based Physical-Activity Promotion at the Workplace: Study Protocol for a Mixed-Methods Study in Germany (KomRueBer Study).

Schaller A; Hoffmann C

2021-06-04

154

Increasing Awareness of the Importance of Physical Activity and Healthy Nutrition: Results From a Mixed-Methods Evaluation of a Workplace Program.

Meyer D; Jayawar MW; Muir S; Ho D; Sackett O

2019-04-01

155

Effects on body weight, eating behavior, and quality of life of a low-energy diet combined with behavioral group treatment of persons with class II or III obesity: A 2-year pilot study.

Karlsson J; Galavazi M; Jansson S; Jendle J

2021-02-01

156

Sustainable Transportation Attitudes and Health Behavior Change: Evaluation of a Brief Stage-Targeted Video Intervention.

Mundorf N; Redding CA; Paiva AL

2018-01-18

157

Optimal Health (Spirit, Mind, and Body): A Feasibility Study Promoting Well-Being for Health Behavior Change.

Walker J; Ainsworth B; Hooker S; Keller C; Fleury J; Chisum J; Swan P

2015-10-01

158

Promoting Employee Health Through an American Cancer Society Program, The CEOs Challenge, Washington State, 2013-2015.

Harris JR; Parrish AT; Kohn M; Hammerback K; McMillan B; Hannon PA

2015-12-17

159

Heart healthy online: an innovative approach to risk reduction in the workplace.

Deitz D; Cook

160

Effectiveness of progressive muscle relaxation therapy as a worksite health promotion program in the automobile assembly line.

Sundram BM; Dahlui M; Chinna K

2016-06-10

161

The Effect of Food Vouchers and an Educational Intervention on Promoting Healthy Eating in Vulnerable Families: A Pilot Study.

Miguel-Berges ML; Jimeno-Martínez A; Larruy-García A; Moreno LA; Rodríguez G; Iguacel I

2022-11-23

162

[The role of physical activity in health promotion of healthcare workers].

Terebessy A; Matyasovszky M; Horváth F; Horosz Á; Juhász I; Győrffy Z

2016-09-01

1

163

Behavioral Phenotyping in Health Promotion: Embracing or Avoiding Failure.

Kangovi S; Asch DA

2018-05-22

164

Promotion of Healthy Aging Within a Community Center Through Behavior Change: Health and Fitness Findings From the AgeWell Pilot Randomized Controlled Trial.

Thom JM; Nelis SM; Cooney JK; Hindle JV; Jones IR; Clare L

2021-02-01

165

The Effect of an Empowerment Program on the Health-promoting Behaviors of Iranian Women Workers: A Randomized Controlled Trial.

Noori F; Behboodimoghadam Z; Haghani S; Pashaeypoor S

2021-07-01

166

Participation effects of workplace promoting activities on healthy eating behavior.

Lord AYZ; Chia

167

Effectiveness of a Blended Web-Based Intervention to Raise Sleep Awareness at Workplace: The WarmUapp™ Pilot Study.

Montagni I; Dehman A; Yu Z; Martinez MJ; Banner S; Rimbert S; Hayez S; Foster C; Fontvieille AM

2019-06-01

168

Participatory action research in corrections: The HITEC 2 program.

Cherniack M; Dussetschleger J; Dugan A; Farr D; Namazi S; El Ghaziri M; Henning R

2016-03-01

169

Creative approach for successful aging: A pilot study of an intergenerational health promotion program.

Lin YC; Dai YT; Huang LH; Wang SC; Huang GS

2017-11-01

170

Moving On!: A Transition Program for Promoting Healthy Eating and an Active Lifestyle Among Student-Athletes After College.

Shriver LH; Reifsteck EJ; Brooks D

2019-01-01

171

Development and piloting of a Checklist for healthy eating And Physical Activity in the Workplace (CEPAW).

Freak-Poli R; Brand M; Boelsen-Robinson T; Huse O; de Courten M; Peeters A

2021-03-12

172

Interventional Effects of Weight-Loss Policy in a Healthy City among Participants with Metabolic Syndrome.

Tai HC; Tzeng IS; Liang YC; Liao HH; Su CH; Kung WM

2019-01-24

Não aplicado no trabalho

Não multicentrico

173

Change in well-being amongst participants in a four-month pedometer-based workplace health program.

Freak-Poli RL; Wolfe R; Wong E; Peeters A

2014-09-15

174



Differences in eating and body-related attitudes, beliefs and behaviors among female graduate students in nutrition and dietetics and naturopathic medicine: a pilot study.

Harris C

175

Psychological and behavioral effects of obesity prevention campaigns.

Simpson CC; Griffin BJ; Mazzeo SE

2019-08-01

176

Early Workplace Intervention to Improve the Work Ability of Employees with Musculoskeletal Disorders in a German University Hospital-Results of a Pilot Study.

Schwarze M; Egen C; Gutenbrunner C; Schriek S

2016-09-07

177

Longitudinal analysis of virtual community perceptions of cohesion: The role of cooperation, communication, and competition.

Lyles AA; Loomis C; Mama SK; Siddiqi S; Lee RE

2018-11-01

178

Evaluation of an Integrated Health Promotion Program for a low-income urban population: Findings and lessons learned.

Weiss L; Quint E; Leto C; Vaughn I; Redrovan A; Fernandes M; Lamourt K; Edgar C; Reso A

2021-07-01

179

Public Health Nurses Promoting Healthy Lifestyles (PHeeL-PHiNe): methodology and feasibility.

Polak R; Const

180

Dietary and exercise change following acute cardiac syndrome onset: A latent class growth modelling analysis.

Bennett P; Gruszczynska E; Marke V

2016-10-01

181

Development of a Multicomponent Intervention to Initiate Health Behavior Change in Primary Care: The Kickstart Health Program.

Clark-Sienkiewicz SM; Caño A; Zeman LL; Lumley MA; Gothe N

2021-12-01

182

Targeted Health Behavior Interventions Promoting Physical Activity: A Conceptual Model.

Morgan PJ; Young MD; Smith JJ; Lubans DR

2016-04-01

183

The effect of educational intervention on promoting safe behaviors in textile workers.

Hatami F; Kakavand R

2022-09-01

1

184

Effect of a Multicomponent Intervention Program on Community-Dwelling People With Intellectual Disabilities.

Kim YS; Moon JH; Hong BK; Ho SH

2020-08-01

185

The Effects of a "Health at Every Size(®)"-Based Approach in Obese Women: A Pilot-Trial of the "Health and Wellness in Obesity" Study.

Ulian MD; Be

186

Toward Health Exercise Behavior Change for Teams Using Lifelog Sharing Models.

Nishiyama Y; Okoshi T; Yonezawa T; Nakazawa J; Takashio K; Tokuda H

2016-05-01

187

An exercise in nostalgia: Nostalgia promotes health optimism and physical activity.

Kersten M; Cox CR; Van Enkevort EA

2016-10-01

188

Effects of a nutritional intervention program based on the self-determination theory and promoting the Mediterranean diet.

Leblanc V; Bég

189

The effect of intervention based on Health Action Model to promote workers' safe behavior in Isfahan Steel Company.

Mazaheri MA; Heidarnia A

2015-01-01

190

Use of health promotion manga to encourage physical activity and healthy eating in Japanese patients with metabolic syndrome: a case study.

Shimazaki T; Matsushita M; Iio M; Takenaka K

2018-01-01

191

The development and validation of a new survey tool: the first step to profiling New Zealanders' eating styles and moving patterns.

Maclaren O; Mackay L; Schofield G; Zinn C

2016-08-01

192

Multicomponent positive psychology intervention for health promotion of Brazilian retirees: a quasi-experimental study.

Durgante H; Dell'Aglio DD

2019-02-28

193

Effectiveness of worksite wellness programs based on physical activity to improve workers' health and productivity: a systematic review.

Marin-Farrona M; Wipfli B; Thosar SS; Colino E; Garcia-Unanue J; Gallardo L; Felipe JL; López-Fernández J

2023-05-24

194

Return on investment of workplace-based prevention interventions: a systematic review.

Thonon F; Godon-Rensonnet AS; Perozziello A; Garsi JP; Dab W; Emsalem P

2023-08-01

195

The MedXFit-study - CrossFit as a workplace health intervention: a one-year, prospective, controlled, longitudinal, intervention study.

Brandt T; Heinz E; Klaaßen Y; Limbara S; Mörsdorf M; Schinköthe T; Schmidt A

2024-01-01

196

Workplace-based primary prevention intervention reduces incidence of hypertension: a post hoc analysis of cluster randomized controlled study.

Hu Z; Wang X; Hong C; Zheng C; Zhang L; Chen Z; Zhou H; Tian Y; Cao X; Cai J; Gu R; Tian Y; Shao L; Wang Z

2023-06-14

197

Does a 40-week football and Zumba exercise intervention influence self-reported job satisfaction, work role functioning and sick leave among female hospital employees? A cluster-randomised controlled trial.

Barene S; Krustrup P; Holtermann A

2023-08-01

198

Advantages and disadvantages of mobile applications for workplace health promotion: A scoping review

Junker, M.;

199

The safety and health improvement: enhancing law enforcement departments study: feasibility and findings.

Kuehl KS; Elliot DL; Goldberg L; MacKinnon DP; Vila BJ; Smith J; Miočević M; O'Rourke HP; Valente MJ; DeFrancesco C; Sleigh A; McGinnis W

2014-01-01

200

Fill in our survey to help foster a culture of health and fitness.

Power B

2014-08-26

201

Keys to embracing aging: A healthy aging intervention.

Kostelic AF; Yelland E; Smith A; Shuman C; Cless A

2023-07-01

202

The 'Goldilocks Principle': designing physical activity at work to be 'just right' for promoting health.

Straker L; Mathiassen SE; Holtermann A

2018-07-01

203

Editorial: Understanding the Interaction Between Physical Activity and Diet for the Promotion of Health and Fitness.

Koehler K; Drenowatz C

2021-01-01

204

Behavior Change Fast and Slow: Changing Multiple Key Behaviors a Long-Term Proposition?

2015-11-03

205

Feeling younger at heart may help you live longer. A youthful attitude may lead to better eating and exercising habits.

2015-04-01

206

Eating Together at the Firehouse: How Workplace Commensality Relates to the Performance of Firefighters.

Kniffin KM; Wansink B; Devine CM; Sobal J

2015-08-08

207

Do No Harm: Moving Beyond Weight Loss to Emphasize Physical Activity at Every Size.

Dollar E; Berman M; Adachi-Mejia AM

2017-04-20

208

Correction to: Vitale, Bianchi, Rapetti, Pepe, Giacco, Giacco and Riccardi, A nutritional intervention programme at a worksite canteen to promote a healthful inspired by the traditional Mediterranean diet.

2018-09-01

209

Time-Restricted Eating to Improve Health-A Promising Idea in Need of Stronger Clinical Trial Evidence.

Bhasin S

2022-09-01

210

Early Life Nutrition: Does It Program Childhood Eating Attitudes?

Chapman DJ

2014-08-01

**211**

Fatigue and its management in the workplace

Caldwell, J.A.; Caldwell, J.L.; Thompson, L.A.; Lieberman, H.R.

2019-01-01

**212**

Insomnia interventions among shift workers: An RCT trial in occupational health services

Järnefelt, H.; Härmä, M.; Sallinen, M.; Paajanen, T.; Virkkala, J.; Martimo, K.-P.; Hublin, C.

2019-01-01

**213**

Work and Female Breast Cancer: The State of the Evidence, 2002-2017

Engel, C.L.; Sharima Rasanayagam, M.; Gray, J.M.; Rizzo, J.

2018-01-01

**214**

Occupational safety across jobs and shifts in emergency departments in Denmark

Kirkegaard, M.L.; Kines, P.; Nielsen, H.B.; Garde, A.H.

2018-01-01

**215**

Breast cancer screening among shift workers: a nationwide population-based survey in Korea

Son, H.; Kang, Y.

2017-01-01

**216**

Prevention of fatigue and insomnia in shift workers-a review of non-pharmacological measures

Richter, K.; Acker, J.; Adam, S.; Niklewski, G.

2016-01-01

**217**

Effects of physical activity programmes in the workplace (PAPW) on the perception and intensity of musculoskeletal pain experienced by garment workers.

Pereira, Cynara Cristina Domingues Alves; López, Ramón Fabian Alonso; Vilarta, Roberto

2013-02-01

**218**

Unveiling the Role of the Work Environment in the Quality of Life of Menopausal Physicians and Nurses

Bapayeva, G.; Terzic, M.; Semenova, Y.; Sarria-Santamera, A.; Gusmanov, A.; Aimagambetova, G.; Laganà, A.S.; Chiantera, V.; Kadroldinova, N.; Ukybassova, T.; Kongrtay, K.; Abdukassimova, M.; Togyzbayeva, K.; Terzic, S.

2023-01-01

**219**

Impacts of heavy smoking and alcohol consumption on workplace presenteeism A cross-sectional study

Lee, S.Y.; Lee, J

220

Work and Female Breast Cancer: The State of the Evidence, 2002-2017

Engel, C.L.; Sharima Rasanayagam, M.; Gray, J.M.; Rizzo, J.

2018-01-01

221

Occupational safety across jobs and shifts in emergency departments in Denmark

Kirkegaard, M.L.; Kines, P.; Nielsen, H.B.; Garde, A.H.

2018-01-01

222

Breast cancer screening among shift workers: a nationwide population-based survey in Korea

Son, H.; Kang, Y.

2017-01-01

223

Prevention of fatigue and insomnia in shift workers-a review of non-pharmacological measures

Richter, K.; Acker, J.; Adam, S.; Niklewski, G.

2016-01-01

224

Reducing Risks to Women Linked to Shift Work, Long Work Hours, and Related Workplace Sleep and Fatigue Issues

Caruso, C.C.

2015-01-01

225

Professional stress and burnout syndrome during the Covid pandemic in the medical field

Slaveykov, K.S.; Stoyanov, V.K.; Trifonova, K.Z.

2023-01-01

226

Moderate-intensity aerobic exercise as an adjunct intervention to improve sleep quality among rotating shift nurses

Okechukwu, C.E.; Masala, D.; D'Ettorre, G.; La Torre, G.

2022-01-01

227

A Systematic Review of Workplace-Based Employee Health Interventions and Their Impact on Sleep Duration Among Shift Workers

Robbins, R.; Underwood, P.; Jackson, C.L.; Jean-Louis, G.; Madhavaram, S.; Kuriakose, S.; Vieira, D.; Buxton, O.M.

2021-01-01

228

Cigarette smoking at workplace among resident physicians and nurses in Mansoura University Hospital

Abou-ElWafa, H.S.; Zoromba, M.A.; El-Gilany, A.-H.

2021-01-01

229

Sleepiness and injury risk in emergency medical service workers in Taiwan

Lin, M.-H.; Huang, Y.-C.; Chen, W.-K.; Wang, J.-Y.

2020-01-01

230

Association between health literacy and behavior regarding health checkups and health counseling in Japanese employees: A comprehensive health literacy survey of a Japanese railway company

Kimura, N.; Obara, K.; Akibayashi, N.; Miyamoto, T.

2019-01-01

Não multicentrico

Upload Full text

231

Outcome-based and Participation-based Wellness Incentives: Impacts on Program Participation and Achievement of Health Improvement Targets.

Barleen NA; Marzec ML; Boerger NL; Moloney DP; Zimmerman EM; Dobro J

2017-03-01

232

A Structured Health Intervention for Truckers (SHIFT): A Process Evaluation of a Pilot Health Intervention in a Transport Company.

Varela-Mato V; Caddick N; King JA; Yates T; Stensel DJ; Nimmo MA; Clemes SA

2018-04-01

233

Attitudes Surrounding a Community-Based Fitness Intervention at an Urban FQHC.

Foster K; Stoeckle J; Silverio A; Castellan C; Hogue A; Gouch A; Weinstein L

2019-07-01

234

A Cross-Disciplinary Successful Aging Intervention and Evaluation: Comparison of Person-to-Person and Digital-Assisted Approaches.

Hsu HC; Kuo T; Lin JP; Hsu WC; Yu CW; Chen YC; Xie WZ; Hsu WC; Hsu YL; Yu MT

2018-05-04

235

A Worksite Weight Loss Program: An Innovative Way to Improve Obesity.

Mack A

2021-11-01

236

Evaluation of a successful aging promotion intervention program for middle-aged adults in Taiwan.

Hsu HC; Chuang SH; Hsu SW; Tung HJ; Chang SC; Lee MM; Wang JY; Kuo LT; Tseng FY; Po AT

2019-03-01

237

A 7-Step Strategy for the Implementation of Worksite Lifestyle Interventions: Helpful or Not?

Wierenga D; Engbers LH; Van Empelen P; van Mechelen W

2016-05-01

238

Impact Model-Based Physical-Activity Promotion at the Workplace: Study Protocol for a Mixed-Methods Study in Germany (KomRueBer Study).

Schaller A; Hoffmann C

2021-06-04

239

Increasing Awareness of the Importance of Physical Activity and Healthy Nutrition: Results From a Mixed-Methods Evaluation of a Workplace Program.

Meyer D; Jayawar MW; Muir S; Ho D; Sackett O

2019-04-01

240

Effects on body weight, eating behavior, and quality of life of a low-energy diet combined with behavioral group treatment of persons with class II or III obesity: A 2-year pilot study.

Karlsson J; Galavazi M; Jansson S; Jendle J

2021-02-01

241

Sustainable Transportation Attitudes and Health Behavior Change: Evaluation of a Brief Stage-Targeted Video Intervention.

Mundorf N; Redding CA; Paiva AL

2018-01-18

Upload Full text

242

The Effect of Food Vouchers and an Educational Intervention on Promoting Healthy Eating in Vulnerable Families: A Pilot Study.

Miguel-Berges ML; Jimeno-Martínez A; Larruy-García A; Moreno LA; Rodríguez G; Iguacel I

2022-11-23

243

[The role of physical activity in health promotion of healthcare workers].

Terebessy A; Matyasovszky M; Horváth F; Horosz Á; Juhász I; Győrffy Z

2016-09-01

1

244

Behavioral Phenotyping in Health Promotion: Embracing or Avoiding Failure.

Kangovi S; Asch DA

2018-05-22

245

Promotion of Healthy Aging Within a Community Center Through Behavior Change: Health and Fitness Findings From the AgeWell Pilot Randomized Controlled Trial.

Thom JM; Nelis SM; Cooney JK; Hindle JV; Jones IR; Clare L

2021-02-01

246

The Effect of an Empowerment Program on the Health-promoting Behaviors of Iranian Women Workers: A Randomized Controlled Trial.

Noori F; Behboodimoghadam Z; Haghani S; Pashaeypoor S

2021-07-01

247

Participation effects of workplace promoting activities on healthy eating behavior.

Lord AYZ; Chiang YT; Cheng YY; Chang YP; Chen HJ; Huang YC; Pan WH

2022-12-01

248

Effectiveness of a Blended Web-Based Intervention to Raise Sleep Awareness at Workplace: The WarmUapp™ Pilot Study.

Montagni I; Dehman A; Yu Z; Martinez MJ; Banner S; Rimbert S; Hayez S; Foster C; Fontvieille AM

2019-06-01

249

Participatory action research in corrections: The HITEC 2 program.

Cherniack M; Dussetschleger J; Dugan A; Farr D; Namazi S; El Ghaziri M; Henning R

2016-03-01

250

Creative approach for successful aging: A pilot study of an intergenerational health promotion program.

Lin YC; Dai YT; Huang LH; Wang SC; Huang GS

2017-11-01

251

Moving On!: A Transition Program for Promoting Healthy Eating and an Active Lifestyle Among Student-Athletes After College.

Shriver LH; Reifsteck EJ; Brooks D

2019-01-01

252

Development and piloting of a Checklist for healthy eating And Physical Activity in the Workplace (CEPAW).

Freak-Poli R; Brand M; Boelsen-Robinson T; Huse O; de Courten M; Peeters A

2021-03-12

Upload Full text

253

Interventional Effects of Weight-Loss Policy in a Healthy City among Participants with Metabolic Syndrome.

Tai HC; Tzeng IS; Liang YC; Liao HH; Su CH; Kung WM

2019-01-24

Não aplicado no trabalho

Não multicentrico

254

Change in well-being amongst participants in a four-month pedometer-based workplace health program.

Freak-Poli RL; Wolfe R; Wong E; Peeters A

2014-09-15

255

Differences in eating and body-related attitudes, beliefs and behaviors among female graduate students in nutrition and dietetics and naturopathic medicine: a pilot study.

Harris C

2018-06-01

256

Psychological and behavioral effects of obesity prevention campaigns.

Simpson CC; Griffin BJ; Mazzeo SE

2019-08-01

257

Early Workplace Intervention to Improve the Work Ability of Employees with Musculoskeletal Disorders in a German University Hospital-Results of a Pilot Study.

Schwarze M; Egen C; Gutenbrunner C; Schriek S

2016-09-07

258

Longitudinal analysis of virtual community perceptions of cohesion: The role of cooperation, communication, and competition.

Lyles AA; Loomis C; Mama SK; Siddiqi S; Lee RE

2018-11-01

259

Evaluation of an Integrated Health Promotion Program for a low-income urban population: Findings and lessons learned.

Weiss L; Quint E; Leto C; Vaughn I; Redrovan A; Fernandes M; Lamourt K; Edgar C; Reso A

2021-07-01

Upload Full text

260

Testing the effectiveness of ecolabels to reduce the environmental impact of food purchases in worksite cafeterias: A randomised controlled trial.

Pechey R; Bateman PA; Cook B; Potter C; Clark M; Stewart C; Piernas C; Jebb SA

2022-12-01

261

Effectiveness of a Worksite-Based Lifestyle Intervention on Employees' Obesity Control and Prevention in China: A Group Randomized Experimental Study.

Kong J; Chen Y; Zheng Y; Zhu L; Chen B; Cheng X; Song M; Patrick DL; Beresford SAA; Wang H

2022-05-31

262

Field Test of an m-Health Worksite Health Promotion Program to Increase Physical Activity in Taiwanese Employees: A Cluster-Randomized Controlled Trial.

Huang SJ; Hung WC; Shyu ML; Chou TR; Chang KC; Wai JP

2023-01-01

263

Intervention of physical exercise in the workplace on work ability, depression, anxiety and job satisfaction in workers with sedentary tasks.

Díaz-Benito VJ; Moro MIB; Vanderhaegen F; Remón ÁLC; Lozano JAS; Fernández-Pola EC; Pérez JPH

2022-01-01

264

Effects of worksite exercise intervention (PRODET®) on well-being at work and capability in performing work-related sedentary tasks: A pilot study.

Díaz-Benito VJ; Barriopedro Moro MI; Clemente Remón ÁL; Santacruz Lozano JA; Hervás Pérez JP; Vanderhaegen F

2022-01-01

265

Effectiveness of a worksite lifestyle intervention to reduce BMI among farmworkers in California: a cluster randomised controlled trial.

Matias SL; Riden HE; Lee DS; Bang H; Schenker MB

2022-09-01

266

Cost and cost-effectiveness of the 'Stand and Move at Work' multicomponent intervention to reduce workplace sedentary time and cardiometabolic risk.

Michaud TL; Yo

267

What Could Highly Engaged Workers Gain From Mental Health Promotion Programs?: An Exploratory Analysis of Secondary Outcomes of Brief Daily Workplace Well-being Programs.

Xie W; Ng SM; Wang M; Li HY; Emery C; Lo HHM; Yeung A; Young DKW

2023-03-01

268

Testing the effectiveness of ecolabels to reduce the environmental impact of food purchases in worksite cafeterias: A randomised controlled trial.

Pechey R; Bate

269

Effect of an education-based workplace intervention (move in office with education) on sedentary behaviour and well-being in desk-based workers: a cluster randomized controlled trial.

Patel AK; Banga C; Chandrasekaran B

2022-09-01

270

Methodological approach for measuring the effects of organisational-level interventions on employee withdrawal behaviour.

Akerstrom M; Severin J; Imberg H; Jonsdottir IH; Björk L; Corin L

2021-10-01

271

The effect of messaging on the acceptance of swaps to reduce the energy content of snacks and non-alcoholic drinks ordered in an experimental online workplace canteen: A randomised controlled trial.

Breathnach S; Koutoukidis DA; Lally P; Boniface D; Sutherland A; Llewellyn CH

2021-07-01

272

A cluster-randomized trial of workplace ergonomics and neck-specific exercise versus ergonomics and health promotion for office workers to manage neck pain - a secondary outcome analysis.

Johnston V; Chen X; Welch A; Sjøgaard G; Comans TA; McStea M; Straker L; Melloh M; Pereira M; O'Leary S

2021-01-12

273

Using an e-Health Intervention to Reduce Prolonged Sitting in UK Office Workers: A Randomised Acceptability and Feasibility Study.

Carter SE; Draijer R; Maxwell JD; Morris AS; Pedersen SJ; Graves LEF; Thijssen DHJ; Hopkins ND

2020-12-01

274

Effects of a work-related stress model based mental health promotion program on job stress, stress reactions and coping profiles of women workers: a control groups study.

Ornek OK; Esin

275

Health Promotion Programs and Policies in the Workplace: An Exploratory Study With Alaska Businesses.

Sawchuk CN; Russo J; Ferguson G; Williamson J; Sabin JA; Goldberg J; Madesclaire O; Bogucki OE; Buchwald D

2020-10-15

276

The effectiveness of multi-component interventions targeting physical activity or sedentary behaviour amongst office workers: a three-arm cluster randomised controlled trial.

Nooijen CFJ; Blom V; Ekblom Ö; Heiland EG; Larisch LM; Bojsen-Møller E; Ekblom MM; Kallings LV

2020-09-01

277

The effects of "Workplace Health Promotion Program" in nurses: A randomized controlled trial and one-year follow-up.

Akyurek G; Avci N; Ekici G

2022-09-01

278

"Oh-oh, the others are standing up... I better do the same". Mixed-method evaluation of the implementation process of 'Take a Stand!' - a cluster randomized controlled trial of a multicomponent intervention to reduce sitting time among office workers.

Danquah IH; Kloster S; Tolstrup JS

2020-08-08

279

Polygenic risk score for obesity and the quality, quantity, and timing of workplace food purchases: A secondary analysis from the ChooseWell 365 randomized trial.

Dashti HS; Hivert MF; Levy DE; McCurley JL; Saxena R; Thorndike AN

2020-07-01

280

A blended intervention to promote physical activity, health and work productivity among office employees using intervention mapping: a study protocol for a cluster-randomized controlled trial.

Sun Y

281

Results of caring and reaching for health (CARE): a cluster-randomized controlled trial assessing a worksite wellness intervention for child care staff.

Linnan LA; Vaughn AE; Smith FT; Westgate P; Hales D; Arandia G; Neshteruk C; Willis E; Ward DS

2020-05-15

282

A Combined Health Action Process Approach and mHealth Intervention to Increase Non-Sedentary Behaviours in Office-Working Adults-A Randomised Controlled Trial.

Rollo S; Prapavessis H

2020-11-01

283

Feasibility and preliminary effects of a peer-led motivationally-embellished workplace walking intervention: A pilot cluster randomized trial (the START trial).

Thøgersen-Ntoumani C; Quested E; Smith BS; Nicholas J; McVeigh J; Fenton SAM; Stamatakis E; Parker S; Pereira G; Gucciardi DF; Ntoumanis N

2020-04-01

284

How Financial Incentives Increase Smoking Cessation: A Two-Level Path Analysis.

van den Brand FA; Candel MJJM; Nagelhout GE; Winkens B; van Schayck CP

2021-01-07

285

Effectiveness of the multi-component dynamic work intervention to reduce sitting time in office workers - Results from a pragmatic cluster randomised controlled trial.

Renaud LR; Jelsma JGM; Huysmans MA; van Nassau F; Lakerveld J; Speklé EM; Bosmans JE; Stijnman DPM; Loyen A; van der Beek AJ; van der Ploeg HP

2020-04-01

286

Sedentary Behaviour and Diabetes Information as a Source of Motivation to Reduce Daily Sitting Time in Office Workers: A Pilot Randomised Controlled Trial.

Rollo S; Prapavessis H

2020-07-01

287

Health-Promoting Effects of a Concurrent Workplace Training Program in Inactive Office Workers (HealPWorkers): A Randomized Controlled Study.

Karatrantou K; Gerodimos V; Manouras N; Vasilopoulou T; Melissopoulou A; Mesiakaris AF; Theodorakis Y

2020-05-01

289

Self-efficacy and Physical Activity in Overweight and Obese Adults Participating in a Worksite Weight Loss Intervention: Multistate Modeling of Wearable Device Data.

Robertson MC;

290

Economic Evaluation of an Intervention Promoting Adoption of Occupational Sun Protection Policies.

Meenan RT; Walkosz BJ; Buller DB; Eye R; Buller MK; Wallis AD; Olivas S

2019-12-01

291

Workplace-Based Exercise Intervention Improves Work Ability in Office Workers: A Cluster Randomised Controlled Trial.

Ting JZR; Chen X; Johnston V

2019-07-24

292

Effect of a participatory organizational workplace intervention on workplace social capital: post-hoc results from a cluster randomized controlled trial.

Framke E; Sørensen OH; Pedersen J; Clausen T; Borg V; Rugulies R

2019-06-06

293

Tailored feedback reduced prolonged sitting time and improved the health of housewives: a single-blind randomized controlled pilot study.

Kitagawa T; Higuchi Y; Todo E; Ueda T; Ando S; Murakami T

2020-02-01

294

Socio-Ecological Natural Experiment with Randomized Controlled Trial to Promote Active Commuting to Work: Process Evaluation, Behavioral Impacts, and Changes in the Use and Quality of Walking and Cycling Paths.

Aittasalo M; Tiilikainen J; Tokola K; Suni J; Sievänen H; Vähä-Ypyä H; Vasankari T; Seimelä T; Metsäpuro P; Foster C; Titze S

2019-05-13

295

Evaluation of a policy intervention to promote the health and wellbeing of workers in small and medium sized enterprises - a cluster randomised controlled trial.

2019-05-02

296

A Cluster Randomized Controlled Trial to Evaluate HeadCoach: An Online Mental Health Training Program for Workplace Managers.

Gayed A; Brya

297

Design of a randomized trial testing a multi-level weight-control intervention to reduce obesity and related health conditions in low-income workers.

Stein RI; Strickland JR; Tabak RG; Dale AM; Colditz GA; Evanoff BA

2019-04-01

298

[Effects of an Integrated Physical Activity Program for Physically Inactive Workers: Based on the PRECEDE-PROCEED Model].

Kim HJ; Choo J

2018-12-01

1

299

Implementation of Occupational Sun Safety at a 2-Year Follow-Up in a Randomized Trial: Comparison of Sun Safe Workplaces Policy Intervention to Attention Control.

Buller DB; Walkosz BJ; Buller MK; Wallis A; Andersen PA; Scott MD; Meenan RT; Cutter GR

2019-06-01

300

Effects of a Classroom Training Program for Promoting Health Literacy Among IT Managers in the Workplace: A Randomized Controlled Trial.

Fiedler S; Pfaff H; Petrowski K; Pförtner TK

2019-01-01

301

Employees' and line managers' active involvement in participatory organizational interventions: Examining direct, reversed, and reciprocal effects on well-being.

Tafvelin S; von Thiele Schwarz U; Nielsen K; Hasson H

2019-02-01

verificar desfecho

302

Combined Before-and-After Workplace Intervention to Promote Healthy Lifestyles in Healthcare Workers (STI-VI Study): Short-Term Assessment.

Scapell

303

A randomized controlled trial of the effect of participatory ergonomic low back pain training on workplace improvement.

Kajiki S; Izumi H; Hayashida K; Kusumoto A; Nagata T; Mori K

2017-05-25

304

A 30-month worksite-based lifestyle program to promote cardiovascular health in middle-aged bank employees: Design of the TANSNIP-PESA randomized controlled trial.

Coffeng JK; van der Ploeg HP; Castellano JM; Fernández-Alvira JM; Ibáñez B; García-Lunar I; van der Beek AJ; Fernández-Ortiz A; Mocoroa A; García-Leal L; Cárdenas E; Rojas C; Martínez-Castro MI; Santiago-Sacristán S; Fernández-Gallardo M; Mendiguren JM; Bansilal S; van Mechelen W; Fuster V

2017-02-01

305

Using nudging and social marketing techniques to create healthy worksite cafeterias in the Netherlands: intervention development and study design.

Velema E; Vyth EL; Steenhuis IH

2017-01-11

Mental health promotion for junior physicians working in emergency medicine: evaluation of a pilot study.

Mache S; Bernburg M; Baresi L; Groneberg D

2018-06-01

306

Impact of Booster Breaks and Computer Prompts on Physical Activity and Sedentary Behavior Among Desk-Based Workers: A Cluster-Randomized Controlled Trial.

Taylor WC; Paxton RJ; Shegog R; Coan SP; Dubin A; Page TF; Rempel DM

2016-11-17

307

Evaluating the effectiveness of organisational-level strategies with or without an activity tracker to reduce office workers' sitting time: a cluster-randomised trial.

Brakenridge CL; Fjeldsoe BS; Young DC; Winkler EA; Dunstan DW; Straker LM; Healy GN

2016-11-04

308

Background, design and conceptual model of the cluster randomized multiple-component workplace study: FRamed Intervention to Decrease Occupational Muscle pain - "FRIDOM".

Christensen JR; Bredahl TV; Hadrévi J; Sjøgaard G; Søgaard K

2016-10-24

309

Physical activity and relaxation in the work setting to reduce the need for recovery: what works for whom?

Formanoy MA; Dusseldorp E; Coffeng JK; Van Mechelen I; Boot CR; Hendriksen IJ; Tak EC

2016-08-24

310

Efficacy of a workplace osteoporosis prevention intervention: a cluster randomized trial.

Tan AM; LaMontagne AD; English DR; Howard P

2016-08-24

311

Exploring the dynamics of a free fruit at work intervention.

Lake AA; Smith SA; Bryant CE; Alinia S; Brandt K; Seal CJ; Tetens I

2016-08-19

Upload Full text

312

Relative benefit of a stage of change approach for the prevention of musculoskeletal pain and discomfort: a cluster randomised trial.

Doda D; Rothmore P; Pisaniello D; Briggs N; Stewart S; Mahmood M; Hiller JE

2015-11-01

313

Worksite Health Program Promoting Changes in Eating Behavior and Health Attitudes.

Mache S; Jensen S; Jahn R; Steudtner M; Ochsmann E; Preuß G

2015-11-01

314

Smoking patterns, quit behaviors, and smoking environment of workers in small manufacturing companies.

Pinsker EA; Hennrikus DJ; Hannan PJ; Lando HA; Brosseau LM

2015-09-01

Creating a Representative Sample of Small Manufacturing Businesses for an Integrated Workplace Safety and Smoking Cessation Intervention Study.

Egelhoff C; Katz M; Brosseau LM; Hennrikus D

2015-07-01

315

Act Healthy: promoting health behaviors and self-efficacy in the workplace.

Schopp LH; Bike DH; Clark MJ; Minor MA

2015-08-01

417

Aerobic exercise reduces biomarkers related to cardiovascular risk among cleaners: effects of a worksite intervention RCT.

Korshøj M; Ravn MH; Holtermann A; Hansen ÅM; Krustrup P

2016-02-01

316

Effects of a workplace intervention on sleep in employees' children.

McHale SM; Lawson KM; Davis KD; Casper L; Kelly EL; Buxton O

2015-06-01

317

Integrating health promotion with quality improvement in a Swedish hospital.

Astnell S; von Thiele Schwarz U; Hasson H; Augustsson H; Stenfors-Hayes T

2016-09-01

318

Does a corporate worksite physical activity program reach those who are inactive? Findings from an evaluation of the Global Corporate Challenge.

Macniven R; Engelen L; Kacen MJ; Bauman A

2015-08-01

319

Employers' views of promoting walking to work: a qualitative study.

Audrey S; Procter S

2015-02-11

320

Effects of a worksite program to improve the cardiovascular health of female health care workers.

Low V; Gebhart B; Reich C

2015-09-01

1

Upload Full text

321

Improvements in Cardiometabolic Risk Factors Among Overweight and Obese Employees Participating in a University Worksite Wellness Program.

Radler DR; Marcus AF; Griehs R; Touger-Decker R

2015-11-01

322

The implementation of multiple lifestyle interventions in two organizations: a process evaluation.

Wierenga D; Engbers LH; Van Empelen P; De Moes KJ; Wittink H; Gründemann R; van Mechelen W

2014-11-01

323

The effect of an e-health intervention designed to reduce prolonged occupational sitting on mean arterial pressure.

Mainsbridge CP; Cooley PD; Fraser SP; Pedersen SJ

2014-11-01

324

A workplace exercise versus health promotion intervention to prevent and reduce the economic and personal burden of non-specific neck pain in office personnel: protocol of a cluster-randomised controlled trial.

Johnston V; O'Leary S; Comans T; Straker L; Melloh M; Khan A; Sjøgaard G

2014-12-01

325

The effectiveness of sit-stand workstations for changing office workers' sitting time: results from the Stand@Work randomized controlled trial pilot.

Chau JY; Daley M; Dunn S; Srinivasan A; Do A; Bauman AE; van der Ploeg HP

2014-10-08

326

Desk-based workers' perspectives on using sit-stand workstations: a qualitative analysis of the Stand@Work study.

Chau JY; Daley M; Srinivasan A; Dunn S; Bauman AE; van der Ploeg HP

2014-07-25

327

Happy@Work: protocol for a web-based randomized controlled trial to improve mental well-being among an Asian working population.

Yuan Q; Liu S; Tang S; Zhang D

2014-07-04

328

A conceptual model for worksite intelligent physical exercise training--IPET--intervention for decreasing life style health risk indicators among employees: a randomized controlled trial.

Sjøgaard G; Justesen JB; Murray M; Dalager T; Søgaard K

2014-06-26

329

Adoption of workplaces and reach of employees for a multi-faceted intervention targeting low back pain among nurses' aides.

Rasmussen CD; Larsen AK; Holtermann A; Søgaard K; Jørgensen MB

2014-05-01

Upload Full text

330

Domain-Specific Active and Sedentary Behaviors in Relation to Workers' Presenteeism and Absenteeism

Koohsari, MJ; Yasunaga, A; McCormack, GR; Shibata, A; Ishii, K; Nakaya, T; Oka, K; Koohsari, Mohammad Javad; Yasunaga, Akitomo; McCormack, Gavin R.; Shibata, Ai; Ishii, Kaori; Nakaya, Tomoki; Oka, Koichiro

2021-10-01

331

The Office Work and Stretch Training (OST) Study: An Individualized and Standardized Approach to Improve the Quality of Life in Office Workers

Holzgreve, F; Maltry, L; Hänel, J; Schmidt, H; Bader, A; Frei, M; Filmann, N; Groneberg, DA; Ohlendorf, D; van Mark, A; Holzgreve, Fabian; Maltry, Laura; Haenel, Jasmin; Schmidt, Helmut; Bader, Andreas; Frei, Markus; Filmann, Natalie; Groneberg, David Alexander; Ohlendorf, Daniela; van Mark, Anke

2020-06-01

332

Model Predictions of Occupational Exposures to Diacetyl and 2,3-Pentanedione Emitted From Roasted Whole Bean and Ground Coffee: Influence of Roast Level and Physical Form on Specific Emission Rates

LeBouf, RF; Ranpara, A; Fernandez, E; Burns, DA; Fortner, AR; LeBouf, Ryan F.; Ranpara, Anand; Fernandez, Elizabeth; Burns, Dru A.; Fortner, Alyson R.

2022-03-23

333

Influence of occupational exposure on hyperuricemia in steelworkers: a nested case-control study

Chen, YY; Yang, YZ; Zheng, ZW; Wang, H; Wang, XL; Si, ZK; Meng, R; Wang, GL; Wu, JH; Chen, Yuanyu; Yang, Yongzhong; Zheng, Ziwei; Wang, Hui; Wang, Xuelin; Si, Zhikang; Meng, Rui; Wang, Guoli; Wu, Jianhui

2022-08-08

334

Risk of symptomatic osteoarthritis associated with exposure to ergonomic factors at work in a nationwide Italian survey

d'Errico, A; Fontana, D; Sebastiani, G; Ardito, C; d'Errico, Angelo; Fontana, Dario; Sebastiani, Gabriella; Ardito, Chiara

2023-01-01

335

Impact of workplace displacement during a natural disaster on computer performance metrics: A 2-year interrupted time series analysis

Sarnosky, K

336

Self-reported sickness absence and presenteeism as predictors of future disability pension: Cohort study with 11-year register follow-up

López-Bueno, R; Clausen, T; Calatayud, J; Bláfoss, R; Vinstrup, J; Andersen, LL; Lopez-Bueno, Ruben; Clausen, Thomas; Calatayud, Joaquin; Blafoss, Runi; Vinstrup, Jonas; Andersen, Lars Louis

2021-07-01

337

UVR Exposure and Prevention of Street Construction Workers in Colombia and Germany

Ruales, MCF; Westerhausen, S; Gallo, HZA; Strehl, B; Guzman, SND; Versteeg, H; Stöppelmann, W; Wittlich, M; Calvache Ruales, Mayra F.; Westerhausen, Stephan; Zapata Gallo, Hernan A.; Strehl, Benjamin; Naza Guzman, Sergio D.; Versteeg, Helmut; Stoppelmann, Wiho; Wittlich, Marc

2022-06-01

338

Disparate exposure to physically demanding working conditions in France

Havet, N; Fournier, J; Stefanelli, J; Plantier, M; Penot, A; Havet, N.; Fournier, J.; Stefanelli, J.; Plantier, M.; Penot, A.

2020-11-01

339

Desk based prompts to replace workplace sitting with stair climbing; a pilot study of acceptability, effects on behaviour and disease risk factors

Azmi, ISMM; Wallis, GA; White, MJ; Puig-Ribera, A; Eves, FF; Azmi, Intan Suhana Munira Mat; Wallis, Gareth A.; White, Mike J.; Puig-Ribera, Anna; Eves, Frank F.

2022-10-31

340

Corrective exercises administered online vs at the workplace for pain and function in the office workers with upper crossed syndrome: randomized controlled trial

Yaghoubitajani, Z; Gheitasi, M; Bayattork, M; Andersen, LL; Yaghoubitajani, Zohreh; Gheitasi, Mehdi; Bayattork, Mohammad; Andersen, Lars Louis

2022-10-01

**341**

Effects of six month personalized endurance training on work ability in middle-aged sedentary women: a secondary analysis of a randomized controlled trial

Stenner, HT; Eigendorf, J; Kerling, A; Kueck, M; Hanke, AA; Boyen, J; Nelius, AK; Melk, A; Boethig, D; Bara, C; Hilfiker, A; Berliner, D; Bauersachs, J; Hilfiker-Kleiner, D; Eberhard, J; Stiesch, M; Schippert, C; Haverich, A; Tegtbur, U; Haufe, S; Stenner, Hedwig T.; Eigendorf, Julian; Kerling, Arno; Kueck, Momme; Hanke, Alexander A.; Boyen, Johanna; Nelius, Anne-Katrin; Melk, Anette; Boethig, Dietmar; Bara, Christoph; Hilfiker, Andres; Berliner, Dominik; Bauersachs, Johann; Hilfiker-Kleiner, Denise; Eberhard, Jorg; Stiesch, Meike; Schippert, Cordula; Haverich, Axel; Tegtbur, Uwe; Haufe, Sven

2020-05-06

**342**

Health and Safety Regulations for COVID-19: A Policy Analysis

Brosseau, LM; Jones, RM; Gardner, K; Williams, SC; Henry, KP; Sanders, D; Brosseau, Lisa M.; Jones, Rachael M.; Gardner, Kate; Williams, Spencer C.; Henry, Kimberly P.; Sanders, Denali

2023-01-12

**343**

Quasi-experimental design for using an interactive social media intervention program to improve truck drivers' health beliefs and eating behaviors

Chang, SL; Wu, WC; Hu, YJ; Lai, HY; Wong, TC; Chang, Ssu-Lan; Wu, Wen-Chi; Hu, Yih-Jin; Lai, Hsin-Yi; Wong, Te-Chih

2022-08-04

1

**344**

New methods for assessing secondary performance attributes of sunscreens suitable for professional outdoor work

Rocholl, M; Weinert, P; Bielfeldt, S; Laing, S; Wilhelm, KP; Ulrich, C; John, SM; Rocholl, Marc; Weinert, Patricia; Bielfeldt, Stephan; Laing, Sabrina; Wilhelm, Klaus Peter; Ulrich, Claas; John, Swen Malte

2021-07-05

**345**

Sit Less and Move More-A Multicomponent Intervention With and Without Height-Adjustable Workstations in Contact Center Call Agents A Pilot Randomized Controlled Trial

Morris, AS; Murphy, RC; Hopkins, ND; Low, DA; Healy, GN; Edwardson, CL; Collins, B; Timpson, H; Shepherd, SO; Cochrane, M; Gavin, D; Graves, LEF; Morris, Abigail S.; Murphy, Rebecca C.; Hopkins, Nicola D.; Low, David A.; Healy, Genevieve N.; Edwardson, Charlotte L.; Collins, Brendan; Timpson, Hannah; Shepherd, Sam O.; Cochrane, Madeleine; Gavin, David; Graves, Lee E. F.

2021-01-01

**346**

The Feasibility of a Text-Messaging Intervention Promoting Physical Activity in Shift Workers: A Process Evaluation

Monnaatsie, M.; Biddle, S.J.H.; Kolbe-Alexander, T.

Date: 2023-01-01

**347**

Workplace exercise program in a hospital environment: an effective strategy for the promotion of employees physical and mental health.

Gerodimos V; Karatrantou K; Papazeti K; Batatolis C; Krommidas C

Date: 2022-09-01

**348**

|  |
| --- |

Changing Diet and Physical Activity in Nurses: A PilotStudy and Process Evaluation Highlighting Challenges in Workplace Health Promotion.

[Luciana Torquati](https://pubmed.ncbi.nlm.nih.gov/?term=Torquati+L&cauthor_id=29650395) [1](https://pubmed.ncbi.nlm.nih.gov/29650395/#full-view-affiliation-1), [Tracy Kolbe-Alexander](https://pubmed.ncbi.nlm.nih.gov/?term=Kolbe-Alexander+T&cauthor_id=29650395) [2](https://pubmed.ncbi.nlm.nih.gov/29650395/#full-view-affiliation-2), [Toby Pavey](https://pubmed.ncbi.nlm.nih.gov/?term=Pavey+T&cauthor_id=29650395) [3](https://pubmed.ncbi.nlm.nih.gov/29650395/#full-view-affiliation-3), [Michael Leveritt](https://pubmed.ncbi.nlm.nih.gov/?term=Leveritt+M&cauthor_id=29650395) [4](https://pubmed.ncbi.nlm.nih.gov/29650395/#full-view-affiliation-4)

2018 Apr 9

**349**

The effectiveness of the Structured Health Intervention For Truckers (SHIFT): a cluster randomised controlled trial (RCT)

[Stacy A. Clemes](https://bmcmedicine.biomedcentral.com/articles/10.1186/s12916-022-02372-7#auth-Stacy_A_-Clemes-Aff1-Aff2), [Veronica Varela-Mato](https://bmcmedicine.biomedcentral.com/articles/10.1186/s12916-022-02372-7#auth-Veronica-Varela_Mato-Aff1-Aff2), [Danielle H. Bodicoat](https://bmcmedicine.biomedcentral.com/articles/10.1186/s12916-022-02372-7#auth-Danielle_H_-Bodicoat-Aff3),

[Cassandra L. Brookes](https://bmcmedicine.biomedcentral.com/articles/10.1186/s12916-022-02372-7#auth-Cassandra_L_-Brookes-Aff4), [Yu-Ling Chen](https://bmcmedicine.biomedcentral.com/articles/10.1186/s12916-022-02372-7#auth-Yu_Ling-Chen-Aff1-Aff2),

[Charlotte L. Edwardson](https://bmcmedicine.biomedcentral.com/articles/10.1186/s12916-022-02372-7#auth-Charlotte_L_-Edwardson-Aff2-Aff5), [Laura J. Gray](https://bmcmedicine.biomedcentral.com/articles/10.1186/s12916-022-02372-7#auth-Laura_J_-Gray-Aff6), [Amber J. Guest](https://bmcmedicine.biomedcentral.com/articles/10.1186/s12916-022-02372-7#auth-Amber_J_-Guest-Aff1), [Vicki Johnson](https://bmcmedicine.biomedcentral.com/articles/10.1186/s12916-022-02372-7#auth-Vicki-Johnson-Aff7), [Fehmidah Munir](https://bmcmedicine.biomedcentral.com/articles/10.1186/s12916-022-02372-7#auth-Fehmidah-Munir-Aff1-Aff2), [Nicola J. Paine](https://bmcmedicine.biomedcentral.com/articles/10.1186/s12916-022-02372-7#auth-Nicola_J_-Paine-Aff1-Aff2), [Gerry Richardson](https://bmcmedicine.biomedcentral.com/articles/10.1186/s12916-022-02372-7#auth-Gerry-Richardson-Aff8),

[Katharina Ruettger](https://bmcmedicine.biomedcentral.com/articles/10.1186/s12916-022-02372-7#auth-Katharina-Ruettger-Aff1),[Mohsen Sayyah](https://bmcmedicine.biomedcentral.com/articles/10.1186/s12916-022-02372-7#auth-Mohsen-Sayyah-Aff1), [Aron Sherry](https://bmcmedicine.biomedcentral.com/articles/10.1186/s12916-022-02372-7#auth-Aron-Sherry-Aff1-Aff2), [Ana Suazo Di Paola](https://bmcmedicine.biomedcentral.com/articles/10.1186/s12916-022-02372-7#auth-Ana_Suazo-Paola-Aff4), [Jacqui Troughton](https://bmcmedicine.biomedcentral.com/articles/10.1186/s12916-022-02372-7#auth-Jacqui-Troughton-Aff7), [Thomas Yates](https://bmcmedicine.biomedcentral.com/articles/10.1186/s12916-022-02372-7#auth-Thomas-Yates-Aff2-Aff5) & [James A. King](https://bmcmedicine.biomedcentral.com/articles/10.1186/s12916-022-02372-7#auth-James_A_-King-Aff1-Aff2)

2022 may 24

**350**

Effectiveness and response differences of a multidisciplinary workplace health promotion program for healthcare workers

[Kai-Hung Cheng](https://pubmed.ncbi.nlm.nih.gov/?term=Cheng+KH&cauthor_id=35957848) , [Ning-Kuang Wu](https://pubmed.ncbi.nlm.nih.gov/?term=Wu+NK&cauthor_id=35957848) , [Chao-Tung Chen](https://pubmed.ncbi.nlm.nih.gov/?term=Chen+CT&cauthor_id=35957848) , [Chih-Yu Hsu](https://pubmed.ncbi.nlm.nih.gov/?term=Hsu+CY&cauthor_id=35957848) , [Yen-An Lin](https://pubmed.ncbi.nlm.nih.gov/?term=Lin+YA&cauthor_id=35957848) , [John Jiin-Chyuan Luo](https://pubmed.ncbi.nlm.nih.gov/?term=Luo+JJ&cauthor_id=35957848) , [Li-Ang Lee](https://pubmed.ncbi.nlm.nih.gov/?term=Lee+LA&cauthor_id=35957848) , [Hai-Hua Chuang](https://pubmed.ncbi.nlm.nih.gov/?term=Chuang+HH&cauthor_id=35957848)

2022 Jul 26

**351**

Worksite neighborhood and obesogenic behaviors: findings among employees in the Promoting Activity and Changes in Eating (PACE) trial.

Barrington WE; Beresford SA; Koepsell TD; Duncan GE; Moudon AV

Date: 2015-01-01

**352**

The effect of complex workplace dietary interventions on employees' dietary intakes, nutrition knowledge and health status: a cluster controlled trial

[Fiona Geaney](https://pubmed.ncbi.nlm.nih.gov/?term=Geaney+F&cauthor_id=27208667) [1](https://pubmed.ncbi.nlm.nih.gov/27208667/#full-view-affiliation-1), [Clare Kelly](https://pubmed.ncbi.nlm.nih.gov/?term=Kelly+C&cauthor_id=27208667) [2](https://pubmed.ncbi.nlm.nih.gov/27208667/#full-view-affiliation-2), [Jessica Scotto Di Marrazzo](https://pubmed.ncbi.nlm.nih.gov/?term=Di+Marrazzo+JS&cauthor_id=27208667) [2](https://pubmed.ncbi.nlm.nih.gov/27208667/#full-view-affiliation-2), [Janas M Harrington](https://pubmed.ncbi.nlm.nih.gov/?term=Harrington+JM&cauthor_id=27208667) [2](https://pubmed.ncbi.nlm.nih.gov/27208667/#full-view-affiliation-2), [Anthony P Fitzgerald](https://pubmed.ncbi.nlm.nih.gov/?term=Fitzgerald+AP&cauthor_id=27208667) [2](https://pubmed.ncbi.nlm.nih.gov/27208667/#full-view-affiliation-2), [Birgit A Greiner](https://pubmed.ncbi.nlm.nih.gov/?term=Greiner+BA&cauthor_id=27208667) [2](https://pubmed.ncbi.nlm.nih.gov/27208667/#full-view-affiliation-2), [Ivan J Perry](https://pubmed.ncbi.nlm.nih.gov/?term=Perry+IJ&cauthor_id=27208667) [2](https://pubmed.ncbi.nlm.nih.gov/27208667/#full-view-affiliation-2)

2016 May 18

**353**

Effectiveness of workplace exercise supervised by a physical therapist among nurses conducting shift work: A randomized controlled trial

[Ryutaro Matsugaki](https://pubmed.ncbi.nlm.nih.gov/?term=%22Matsugaki%20R%22%5BAuthor%5D) 1,2, [Satoshi Kuhara](https://pubmed.ncbi.nlm.nih.gov/?term=%22Kuhara%20S%22%5BAuthor%5D) 1,2, [Satoru Saeki](https://pubmed.ncbi.nlm.nih.gov/?term=%22Saeki%20S%22%5BAuthor%5D) 3, [Ying Jiang](https://pubmed.ncbi.nlm.nih.gov/?term=%22Jiang%20Y%22%5BAuthor%5D) 2, [Ryoma Michishita](https://pubmed.ncbi.nlm.nih.gov/?term=%22Michishita%20R%22%5BAuthor%5D) 2, [Masanori Ohta](https://pubmed.ncbi.nlm.nih.gov/?term=%22Ohta%20M%22%5BAuthor%5D) 4, [Hiroshi Yamato](https://pubmed.ncbi.nlm.nih.gov/?term=%22Yamato%20H%22%5BAuthor%5D) 2

2017 Jun 20

**354**

The SHIELD (Safety & Health Improvement: Enhancing Law Enforcement Departments) Study: Mixed Methods Longitudinal Findings Kerry S. Kuehl, MD, DrPH1, Diane L. Elliot, MD1, David P. MacKinnon, PhD2, Holly P. O’Rourke, MA2, Carol DeFrancesco, MALS1, Milica Miočević, MA2, Matthew Valente, MA2, Adriana Sleigh, BS1, Bharti Garg, MD, MPH1, Wendy McGinnis, MS1, and Hannah Kuehl, MA1

2017 May 01

**355**

Process evaluation of a multifaceted health program aiming to improve physical activity levels and dietary patterns among construction workers.

Viester L; Verhagen EA; Bongers PM; van der Beek AJ

Date: 2014-11-01

**356**

Workplace exercise for changing health behavior related to physical activity.

Grande AJ; Cieslak F; Silva V

Date: 2015-01-01

**357**

A cluster randomized trial of alcohol prevention in small businesses: a cascade model of help seeking and risk reduction.

Reynolds GS; Bennett JB

Date: 2015-01-01

**358**

Effect of a 5-Month Worksite Physical Activity Program on Tertiary Employees Overall Health and Fitness.

Genin PM; Degoutte F; Finaud J; Pereira B; Thivel D; Duclos M

Date: 2017-02-01

**359**

Implementation of an Internet Weight Loss Program in a Worksite Setting.

Ross KM; Wing RR

Date: 2016-01-01

**360**

Improving employee health: evaluation of a worksite lifestyle change program to decrease risk factors for diabetes and cardiovascular disease.

Kramer MK; Molenaar DM; Arena VC; Venditti EM; Meehan RJ; Miller RG; Vanderwood KK; Eaglehouse Y; Kriska AM

**361**

Weight-Dependent Disparities in Adolescent Girls: The Impact of a Brief Pilot Intervention on Exercise and Healthy Eater Identity.

Kramer EN; Chard CA; Walters K; Barr-Anderson DJ

Date: 2018-07-04

**362**

The Impact of a Worksite-Based Diabetes Prevention Intervention: A Pilot Study.

Clark B; Boghani S; Grullon C; Batista M

Date: 2017-06-01

**363**

Promoting physical activity in worksite settings: results of a German pilot study of the online intervention Healingo fit.

Dadaczynski K; Schiemann S; Backhaus O

Date: 2017-09-08

**364**

Does rearranging meal times at night improve cardiovascular risk factors? An Australian pilot randomised trial in night shift workers.

Leung GKW; Davis R; Huggins CE; Ware RS; Bonham MP

2021-06-07

**365**

Association between Dietary Habits, Shift Work, and the Metabolic Syndrome: The Korea Nurses' Health Study.

Jung H; Dan

**366**

Go!: results from a quasi-experimental obesity prevention trial with hospital employees.

LaCaille LJ; S
