# Supplementary material for: The efficacy of interventions in the workplace promoting exercise and a healthy diet among shift workers: A systematic review
Source: PLoS One. 2025 Jun 11;20(6):e0325071. doi: 10.1371/journal.pone.0325071 (PMC12157753; doi:10.1371/journal.pone.0325071)
Supplement: Attachment — Submitted filename: Comments.docx [file pone.0325071.s002.docx]

**Comments:**

The manuscript addresses a valid research question within the journal's scope. Additionally, it explores an important issue regarding the impact of interventions promoting healthy eating and/or physical activity among shift workers.

The title accurately reflects the manuscript's focus and is concise, while the abstract precisely describes the objectives, methods, results, and conclusions.

**To improve the structure and flow of the manuscript, I would like to offer some comments, questions, and suggestions.**

**Abstract:**

- Provide protocol registration information;

- Indicate how risk of bias was assessed.

**Introduction**

- Define the WHO abbreviation upon first mention.

**Materials and Methods**

- Define the PICO strategy, clarifying the Population, Intervention, Control, and Outcomes beyond just identifying the central question;

- It is unnecessary to provide dates in parentheses; I suggest including the dates in the just main text instead;

- Additionally, phrases like "(for instance, sex)" or "(for instance, number of employees)" and similar citations are unclear. It is ambiguous whether only the items in parentheses are considered in the studies or whether additional data are included. I suggest rephrasing: "No limitations were established regarding the gender of the subject, workplace size, number of employees, and [list additional items]."

**Exclusion Criteria**

- Were other study types, such as general reviews (narrative, systematic with or without meta-analysis), preclinical studies, ecological studies, and cost-effectiveness analyses, included? If not, consider stating this explicitly;

- In this section, it states that "studies on youths less than 18 years old" were excluded, yet in the previous section, it mentions "No limitations were established regarding the characteristics of the subject (for instance, sex)." I suggest specifying "evaluated individuals aged ≥ 18 years" to clarify this in the eligibility criteria.

**Evaluation of Bias**

- What about studies that were not RCTs? How were they assessed? I suggest adding an additional evaluation method for them.

**Results**

**Table 1**

- Standardize table borders to the same thickness; for clarity, typically only horizontal lines are used.

Line 1: Describe the exclusion criterion as with other lines; if not already mentioned, please specify;

- Line 2: Describe the inclusion criterion similarly; if not already mentioned, please specify;

- Consider using abbreviations only within the table and including descriptions in a line following the table as a caption;

- Move the Author/Year column to the first position, and instead of placing the reference in superscript with the title, insert the reference in square brackets next to the author's name and publication year, as specified in the author’s guide. For example, in the first line: Monnaat Sie, M et al., 2023 [11];

- Only the last name of the first author is necessary, and a semicolon should follow "et al.," Please review and reformat accordingly.

**Table 2**

- Format the borders similarly to Table 1;

- Include references in square brackets in all rows in column 1;

- Format and standardize italicized items in line 1;

- In the study by Torquati L et al., 2017, correct the text in column 4: "post-intervention anda t 6 months.";

- What are the asterisks in the results in line 1? Please explain;

- Use abbreviations in the table text, with descriptions in a caption for terms such as BMI and FFQ;

- Adjust cell indentation; some spaces are misaligned;

- Specify whether "duration" refers to the intervention or study period to avoid confusion for readers.

**First Paragraph of Results Presentation**

- Consider adding basic data on sample size (n) and percentages, average age, gender proportions, commonly represented countries, and average duration;

- Clarify "RE-AIM structure."

**Line 29, Regarding the Assessment Tool, RoB 2.0**

- For studies where this method was not applied, an alternative risk-of-bias assessment tool should be included so all studies undergo bias evaluation;

- Provide a general conclusion of this analysis, classifying the overall risk of bias in your study as low, medium, or high; this information would be beneficial.

**Discussion**

- The discussion currently presents each study's findings individually; this section should contextualize and connect these findings with the existing literature, incorporating recent references. I recommend restructuring this section;

- Consider presenting these results more succinctly within the results section and using this section to explore common themes that warrant further discussion.

- Include a section on study limitations.

**Conclusion**

- Highlight key findings and potential areas for further research.

**Line Numbering**

- Line numbering should be sequential and continuous, without restarting on each page. I suggest correcting this.

**References**

- I recommend standardizing references throughout the text as specified in the author's guide, using square bracket numbers.
