# Supplementary material for: The efficacy of interventions in the workplace promoting exercise and a healthy diet among shift workers: A systematic review
Source: PLoS One. 2025 Jun 11;20(6):e0325071. doi: 10.1371/journal.pone.0325071 (PMC12157753; doi:10.1371/journal.pone.0325071)
Supplement: Attachment — Submitted filename: Reviewer Md. Al Azim.docx [file pone.0325071.s003.docx]

Manuscript Summary: This study investigates workplace promoting exercise and a healthy diet among shift workers, using Systematic review protocol. The authors conclude that the managers are a way of reinforcing the importance of preventative actions and allows a better adaptation of the organizational context to these activities, there is no single solution. Overall, this study adds valuable insights into workers and their development strategies through exercise and healthy diets.

General Comments:

Contribution to the Field: The study addresses some overcoming context like exercise, and diet habits and its necessity. However, not provide any specific outcome like if this will be taken then it provides much more effective way.

Is the paper well written? Yes, very well written but some sections need much more clarity in accent use.

Specific Comments:

Introduction: In the introduction, the structure needs to be altered. Also, the research gap could be explained more.

Why particularly this day and night shift workers were selected could be explained? In the end of the introduction, how this study could help to solve the problem could be added.

What is the main question addressed by the research?

Methods: Is it relevant and interesting? Very interesting manuscript

How original is the topic? Many groups are studying this topic.

Figures/Tables: Is the text clear and easy to read? Yes, very interesting to read.

Conclusion: Well define.

Recommendations for Improvement:

Please include any additional comments on the tables and figures and quality of the data.The tables and figures are nicely formatted. The way they have added limitations and practical applications make this manuscript pretty unique and clear.

Additional comments:

1. What is the main question addressed by the research? The group is trying to applied for other group of worker like all types of worker? Write it more specifically.
2. What parts do you consider original or relevant for the field? What specific gap in the field does the paper address? A lot of research is going on this exercise and nutrition and its relevant.
3. What specific improvements should the authors consider regarding the methodology? What further study should be considered? Maybe a broader type of sample size of article may be including Scopus index articles.

**Decision Recommendation: Major Revision.**
